# Cucumber CsBPCs Regulate the Expression of *CsABI3* during Seed Germination

**DOI:** 10.3389/fpls.2017.00459

**Published:** 2017-04-03

**Authors:** Ying Mu, Yumei Liu, Longqiang Bai, Shuzhen Li, Chaoxing He, Yan Yan, Xianchang Yu, Yansu Li

**Affiliations:** The Institute of Vegetables and Flowers, Chinese Academy of Agricultural SciencesBeijing, China

**Keywords:** CsBPC, CsABI3, germination, cucumber, transcription factors

## Abstract

Cucumber seeds with shallow dormancy start to germinate in fruit that are harvested late. ABSCISIC ACID INSENSITIVE3 (ABI3), a transcription factor in the abscisic acid (ABA) signaling pathway, is one of the most important regulators in the transition from late embryogenesis to germination. Our analysis found a candidate cis-regulatory motif for cucumber BASIC PENTACYSTEINE (CsBPC) in the promoter of *CsABI3*. Yeast one-hybrid and chromatin immunoprecipitation (ChIP) assays showed that CsBPCs bound to the promoter of *CsABI3*. Examination of β-glucuronidase (GUS) activity driven by the *CsABI3* promoter in transgenic *Arabidopsis thaliana* plants overexpressing *CsBPCs* and a *Nicotiana benthamiana* (tobacco) luciferase assay indicated that CsBPCs inhibited the expression of *CsABI3*. Transgenic plants overexpressing *CsBPCs* were constructed to confirm that CsBPCs participates in the control of seed germination. This study of the cucumber BPC-ABI3 pathway will help to explore and characterize the molecular mechanisms underlying seed germination and will provide necessary information for seed conservation in agriculture and forestry.

## Introduction

Cucumber (*Cucumis sativus L*.), grown worldwide, is a model species of the Cucurbitaceae family. Cucumber is a typical example of fruit with shallow dormancy. During ripening, cucumber seeds start to germinate inside the fruit, also known as pre-harvest sprouting or vivipary, severely damaging the seed quantity and quality. In agriculture, vivipary is a phenomenon that results in worldwide losses in cereal crops, especially in humid regions (Farnsworth, 2000). Seeds undergo a period of dormancy until the proper environment for survival is present. Primary dormancy is generally used to describe an intact freshly-harvested viable seed that cannot germinate in favorable conditions (Bewley, 1997). Warm stratification, chilling (cold stratification), light, or hormones (including gibberellins; Kucera et al., 2005; Miransari and Smith, 2014) can release seeds from primary dormancy. Non-dormant seeds can also re-enter dormancy, known as secondary dormancy, to avoid unfavorable conditions such as season changes (Finkelstein et al., 2008). The breaking of seed dormancy to establish seedling growth is a critical step in the life of seed plants. The regulation of this developmental transition is not only necessary for plant survival but also important for agriculture and forestry; however, the regulatory mechanism underlying remains elusive.

Plant hormones such as Abscisic acid (ABA), gibberellins, brassinosteroids, ethylene, and cytokinins have long been known to participate in the control of seed dormancy and germination (Kucera et al., 2005; Finkelstein et al., 2008; Wang et al., 2011). ABA is a hormone that plays a vital role during the dormancy-to-germination transition (Finkelstein et al., 2002; Nambara and Marion-Poll, 2003; Chen et al., 2008; Kang et al., 2015). ABSCISIC ACID INSENSITIVE3 (ABI3) is an important transcription factor in the ABA signaling pathway (Finkelstein et al., 2008), participating in many different development stages including plastid development (Rohde et al., 2000), bud dormancy and flowering time (Rohde et al., 2002), lateral root development (Brady et al., 2003), desiccation tolerance (Khandelwal et al., 2010), and leaf development (Rohde et al., 1999). In particular, ABI3 is one of the most important regulators in the transition from late embryogenesis to germination (Parcy et al., 1994; Jones et al., 1997; Li and Foley, 1997; Zeng et al., 2003). The *Arabidopsis thaliana* mutant *abi3* had decreased seed storage and a reduction in the level of the late embryogenesis abundant proteins Early methionine-labeled 1 (Em1) and Em6. It was reported that, via ABI3, ABA induced seed maturation behavior including the accumulation of storage substances, the acquisition of desiccation tolerance, and the imposition of dormancy (McCarty et al., 1989; Li and Foley, 1997; Nambara et al., 2000; Raz et al., 2001). Although the transcription level of *ABI3* drops quickly after the breaking of seed dormancy (Park et al., 2011), ABI3 participates in the regulation of seed germination (Parcy et al., 1994; Nambara et al., 2000; Lopezmolina et al., 2002; Bassel et al., 2006; Feng et al., 2014). Given the range of functions that ABI3 is involved in, its expression is strictly regulated. LEAFY COTYLEDON1 (LEC1), LEC2, FUSCA 3 (FUS3), and ABI3 itself are the four important partially redundant transcription factors that regulate expression of *ABI3* in seed development (To et al., 2006). ABI3 is also regulated by ABA during germination (Lopezmolina et al., 2002). During germination and early seedling development, the ABA pathway factor RELATED TO ABI3/VP1 (RAV1) represses the expression of *ABI3, ABI4*, and *ABI5* (Feng et al., 2014). WRKY41 controls seed dormancy via *ABI3* activation (Ding et al., 2014). During seed germination, ABI3 is repressed by the chromatin remodeling factor PICKLE (PKL; Perruc et al., 2007). The BES1-TPL-HDA19 repressor complex also participates in the ABA signaling pathway by controlling the epigenetic silencing of *ABI3* during early seedling development (Ryu et al., 2014). At the protein level, ABI3 is targeted to 26S proteasome degradation by ABI3-INTERACTING PROTEIN 2 (AIP2; Zhang et al., 2005; Zeng et al., 2013), while protein-protein interactions between ABI3 and PIL5 are also responsible for the regulation of ABI3 (Park et al., 2011). Additionally, *ABI3* is regulated by alternative splicing (Sugliani et al., 2010; Gao et al., 2013).

The BASIC PENTACYSTEINE/BARLEY B RECOMBINANT (BPC/BBR) family of transcription factors exists only in the plant kingdom (Sangwan and O'Brian, 2002; Santi et al., 2003; Kooiker et al., 2005; Berger et al., 2011; Simonini and Kater, 2014). While BPCs are known to participate in a wide range of developmental process (Monfared et al., 2011), the mechanism that determines how BPCs are specialized to specific developmental processes remains largely unknown. The barley BBR protein is involved in leaf and flower development, probably by regulating the expression of the homeotic gene *Barley Knox3* (*BKn3*; Santi et al., 2003). The BPC1 protein participated in ovule and embryo development by regulating transcription factors *LEC2, INNER NO OUTER* (*INO)*, and *SEEDSTICK* (*STK*; Meister et al., 2004; Kooiker et al., 2005; Berger et al., 2011). Class I BPCs regulate the inflorescence meristem via the regulation of *SHOOT MERISTEMLESS* (*STM*) and *BREVIPEDICELLUS/KNAT1* (*BP*; Simonini and Kater, 2014).

While the mechanisms underlying seed dormancy have been studied extensively, less is known about the transition from dormancy to germination. ABI3 is one of the most important factors controlling seed germination. In this study, we used yeast one-hybrid and ChIP assays to show that CsBPCs act upstream of CsABI3. We further determined the negative relationship between the CsBPCs and CsABI3 using GUS staining and a luciferase assay. Moreover, the role of cucumber CsBPCs in seed germination was investigated. Analysis of the transcription factors controlling ABI3 during seed germination is useful for understanding the molecular mechanisms underlying seed germination and will provide information for seed conservation in agriculture and forestry.

## Material and methods

### Plant materials and growth conditions

Cucumber (*C. sativus* L.) was used in this study; line 9930 donated by Huang et al. (2009) was used for gene cloning to obtain the target construct and “Xintai Mici” was used for the construction of transgenic plants. *Arabidopsis* (ecotype Columbia) was used as the wild-type and the *bpc1-1 bpc2 bpc4 bpc6* mutant was generously provided by Professor Charles S. Gasser (Monfared et al., 2011).

Cucumber seeds were germinated in darkness at 28°C and then seedlings with two true leaves were transplanted to a growth chamber under a 14-h light (350 μmol m^−2^ s^−1^) at 28°C/10-h dark at 18°C cycle. *Arabidopsis* seeds were surface-sterilized, sown on Murashige and Skoog (MS) medium plus 1.5% sucrose, and then 7-day-old-seedlings were transplanted to a growth chamber at 22°C under a 14-h light (60 μmol m^−2^ s^−1^) /10-h dark cycle.

### Plant transformation

To generate overexpression lines, the coding sequences of *CsBPC1* and *CsBPC3* were amplified and cloned into the *Bam*HI/*Spe*I sites of the pCAMBIA-2300 vector to obtain the Pro35S:CsBPC1-GFP and Pro35S:CsBPC3-GFP constructs.

For *Arabidopsis* plant transformation, constructs were introduced into *Agrobacterium tumefaciens* GV3101. The floral dip method was then used to transform *Arabidopsis* plants with these constructs (Clough and Bent, 1998). The transgenic plants used in this study were selected on plates containing hygromycin or kanamycin and were confirmed by PCR amplification.

For cucumber plant transformation, constructs were transformed into the cucumber pure line Xintai Mici as previously described (Cheng et al., 2015). *Agrobacterium* LB4404 harboring the constructs were used for cucumber transformation. Briefly, cucumber seeds were treated with 70% alcohol for 20 s followed by 3% sodium hypochlorite solution for 7 min before being rinsed five times in sterile deionized water and placed on MS0 medium (MS plus 3% sucrose) for 2–3 days. The basal half of the cotyledon was then harvested and incubated with *Agrobacterium* harboring the target constructs for 15 min. The inoculated explants were transferred onto sterile filter paper and cultured on MS1 medium (MS0 medium plus 0.5 mg L^−1^ 6-Benzylaminopurine and 1 mg L^−1^ ABA) for 2 d in the dark. The explants were incubated on MS1 medium for 15–20 d until the shoot was 1–1.5 cm long. The shoot was then transferred to MS2 (MS plus 200 mg L^−1^ cefotaxime) to develop the root. Integration of the construct in the regenerated plants was confirmed by PCR.

### GFP assays

The coding sequences of the CsBPCs were amplified by RT-PCR and cloned into the *Xho*I/*Eco*RI sites of the transient expression vector PBSK+-35s-EGFP, which contains the *eGFP* coding sequence under the control of the constitutive cauliflower mosaic virus 35S promoter, to generate a CsBPCs fusion protein with GFP at the C-terminus. The *fibrillarin* gene (Sun et al., 2011) was used as a nuclear target control. The resulting constructs were transfected into *Arabidopsis* mesophyll protoplasts according to the method of Yoo et al. (2007). Fluorescence analysis was performed using an OLYMPUS DP72 digital camera.

### GUS activity assays

To generate *ProCsABI3*:*GUS* transgenic plants, the *CsABI3* promoter sequence was amplified and cloned into the *Hind*III/*Nco*I sites upstream of the *GUS* coding sequence in the pCAMBIA1305 plasmid. The resulting construct was transformed into *Arabidopsis* via the *Agrobacterium*-mediated floral dip method (Clough and Bent, 1998). Plants were incubated in GUS staining solution containing 100 mM sodium phosphate buffer (pH 7.2), 0.1% Triton X-100, 10 mM EDTA, 0.5 mM K_4_Fe(CN)_6_·3H_2_O, 0.5 mM K_3_Fe(CN)_6_, and 1 mg L^−1^ X-Gluc at 37°C overnight. Images were taken on a dissecting microscope (OLYMPUS SZX2-ILLB) using a digital camera (Olympus). To quantify the GUS level, *Arabidopsis* seedlings were collected and ground in 100 μL of 1 × Cell Culture Lysis Reagent buffer (Promega), after which the extracts (5 μL) were mixed with 50 μL of luciferase assay substrate (Promega). Then, the extracts (5 μL) were incubated with 50 μL of 4-methylumbelliferyl β-D-glucuronide (MUG) substrate mix (10 mM Tris–HCl, pH 8.0, containing 1 mM MUG and 2 mM MgCl_2_) at 37°C for 30 min, after which the reaction was stopped by adding 945 μL of 0.2 M Na_2_CO3 and the fluorescence intensity was measured using a Modulus Luminometer/Fluorometer and an ultraviolet fluorescence optical kit (Turner Biosystems). GUS activity in each sample is normalized per unit tissue weight.

### Yeast one-hybrid

Yeast one-hybrid assays were performed according to the manufacturer's instructions (Clontech). The *CsABI3* promoter sequence was fused to the *Kpn*I/*Sal*I sites in the pLacZi plasmid (Clontech) that was then linearized by digestion with *Nco*I and integrated into the genome of yeast strain YM4271 to obtain the integrated yeast clone harboring the ProCsABI3-pLacZi construct. To generate CsBPCs-PGAD, the Cs*BPCs* coding sequences were amplified and cloned into *Eco*RI/*Xho*I sites downstream of the GAL4 activation domain coding sequence in PGADT7. CsBPCs-PGAD plasmids or the pGADT7 plasmid alone (negative control) were transformed into the yeast clone harboring the ProCsABI3-pLacZi construct. Transformants were grown on SD/-Ura-Leu dropout plates containing X-gal (5-bromo-4-chloro-3-indolyl-β-D-galactopyranoside) for blue/white colony screening.

### LUC activity assay for DNA-protein interactions in *N. benthamiana* leaves

The reporter construct ProCsABI3:LUC was prepared using the primers listed in Table S1 and the effector constructs Pro35S:CsBPC1-GFP and 35S:CsBPC3-GFP as described above. To generate the ProCsABI3:LUC construct, the promoter sequence of *CsABI3* was PCR-amplified and cloned into the *Hind*III/*Bam*HI sites of the pCAMBIA1305 plasmid.

The reporter and effector constructs were transformed into *Agrobacterium* strain GV3101 to infiltrate *N. benthamiana* leaves as previously reported (Voinnet et al., 2003). The fluorescence intensity was measured under a Modulus Luminometer/Fluorometer using an ultraviolet fluorescence optical kit (Turner Biosystems). The relative LUC levels were expressed as the ratio of LUC to GUS.

### qRT-PCR

Total RNA was isolated using a Qiagen RNeasy Plant Mini Kit and first-strand cDNA was synthesized using the SuperScript® III First-Strand Synthesis System (Invitrogen). PCR was then carried out using the gene-specific primers listed in Table S1 and SYBR PrimeScript Ready Mix (Takara) with an Mx3000p Real-time PCR System (Agilent, Stratagene) according to the manufacturer's instructions. Three biological replicates were included for each sample, and the expression levels were normalized to *Tubulin*.

### ChIP

Cucumber seedlings containing the constructs Pro35S:CsBPC1-GFP and 35S:CsBPC3-GFP were generated as described above and were used to perform the ChIP assay as described by Wang et al. (2002). Transgenic cucumber chromatin was extracted from the GFP-tagged seedlings and then incubated with anti-GFP antibody. The immunoprecipitated DNA was analyzed by qRT-PCR with specific primers listed in Table S1.

### Seed germination rate

To determine the germination rate of cucumber seeds, cucumber fruit were collected 35 d after pollination and the seeds were removed after 5 d of ripening. The seeds were put on Petri dishes (9.0 cm) containing a double layer of filter paper soaked with 5 mL sterilized distilled water and cultured at 28°C for 2 d in the dark. Seeds were considered to have germinated when 3 mm of the radicle had emerged.

For *Arabidopsis*, seeds collected at the same times were stored at room temperature for 40 d before being used. Seeds were sown on MS agar plates to observe germination. The plates were incubated at 23°C under continuous light for 8 d. Germination was scored based on radicle emergence (Liu et al., 2013).

Approximately 50 seeds were used for each replicate for both cucumber and *Arabidopsis*.

## Results

### Cucumber CsBPCs influence seed germination

**Figure 1:**
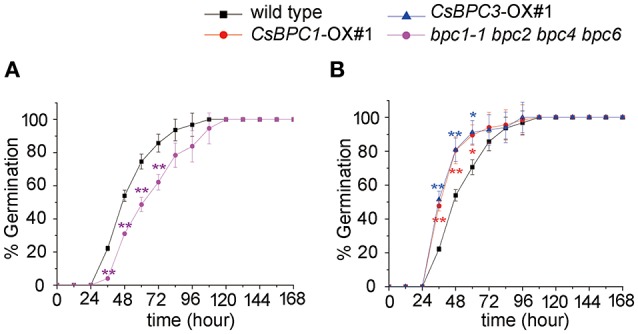
**Germination rate of *Arabidopsis* seeds. (A)** Seed germination rate of the *bpc1-1 bpc2 bpc4 bpc6* mutant and wild-type *Arabidopsis*. **(B)** Seed germination rate of *CsBPC1*-OX#1, *CsBPC3*-OX#1, and wild-type *Arabidopsis*. Germination rates (%) of the seeds were analyzed at the indicated time points. The data represent means ± *SD* of three independent replicates with at least 50 seeds counted per replicate. Values that differed significantly from wild type are indicated. ^*^p < 0.05 and ^**^p < 0.01 by Bonferroni *post-hoc* test.

**Figure 2:**
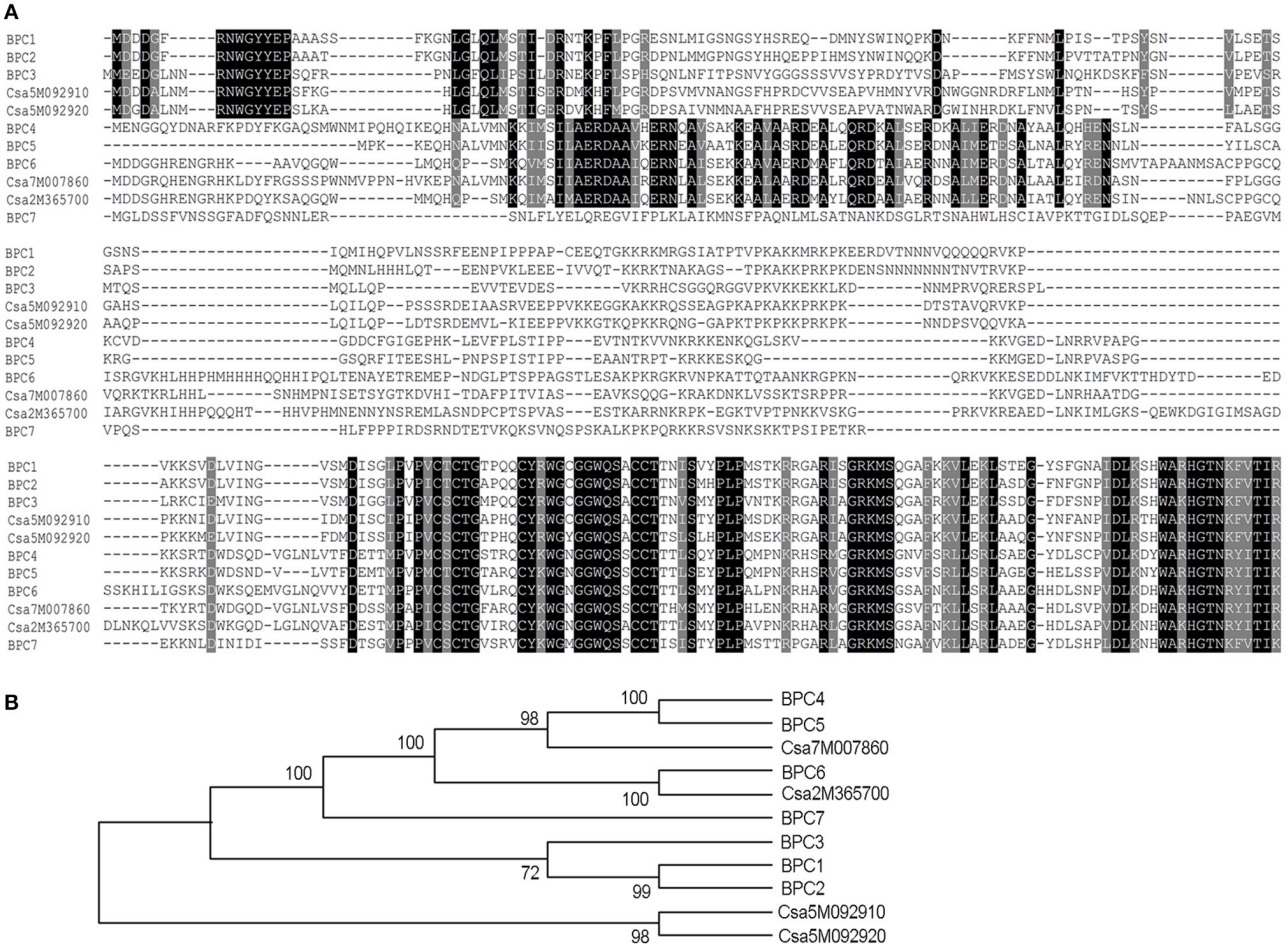
***Arabidopsis* and cucumber BPC homologs. (A)** *Arabidopsis* and cucumber BPC homologs were aligned using CLUSTAL X. Conserved regions between single classes or the whole family are shown in white text on black, with conserved substitutions shaded in gray. Conserved cysteines are marked by an asterisk. **(B)** Phylogeny of the Arabidopsis and Cucumber BPC family. The 11 BPC proteins were classified into three groups using the Neighbor-Joining method (Saitou and Nei, 1987). The optimal tree with the sum of branch length = 2.95032244 is shown. The percentage of replicate trees in which the associated taxa clustered together in the bootstrap test (1,000 replicates) are shown next to the branches. The evolutionary distances were computed using the Poisson correction method and are in the units of the number of amino acid substitutions per site. The analysis involved 11 amino acid sequences. All positions containing gaps and missing data were eliminated; there were a total of 199 positions in the final dataset. Evolutionary analyses were conducted in MEGA7 (Kumar et al., 2016).

In this study, we found that seeds of the *Arabidopsis* quadruple mutant *bpc1-1 bpc2 bpc4 bpc6* exhibited decreased germination rates (Figure 1A). This led us to question whether BPC homologs in cucumber could also affect germination. There are seven BPC proteins (BPC1-BPC7) encoded by the *Arabidopsis* genome. These have been categorized into three classes (Class I-III) based on their sequence similarity (Meister et al., 2004; Monfared et al., 2011). Database (http://www.icugi.org/cgi-bin/ICuGI/index.cgi) searches revealed that four BPC homologs exist in cucumber with highly conserved C-terminal regions. The cucumber BPC homologs were divided into two classes based on amino acid sequence alignments (Meister et al., 2004; Figure 2A) and phylogenic methods (Figure 2B). Class I included CsBPC1 (Csa5M092910) and CsBPC2 (Csa5M092920), while Class II included CsBPC3 (Csa7M007860) and CsBPC4 (Csa2M365700). Like their homologs in other plant species, nuclear fluorescence was observed in the nucleus of *Arabidopsis* protoplast cells expressing the CsBPCs-GFP fusion proteins (Figure S1). For the present study, two members were selected for further analysis, CsBPC1 and CsBPC3, representing Class I and Class II, respectively.

**Figure 3:**
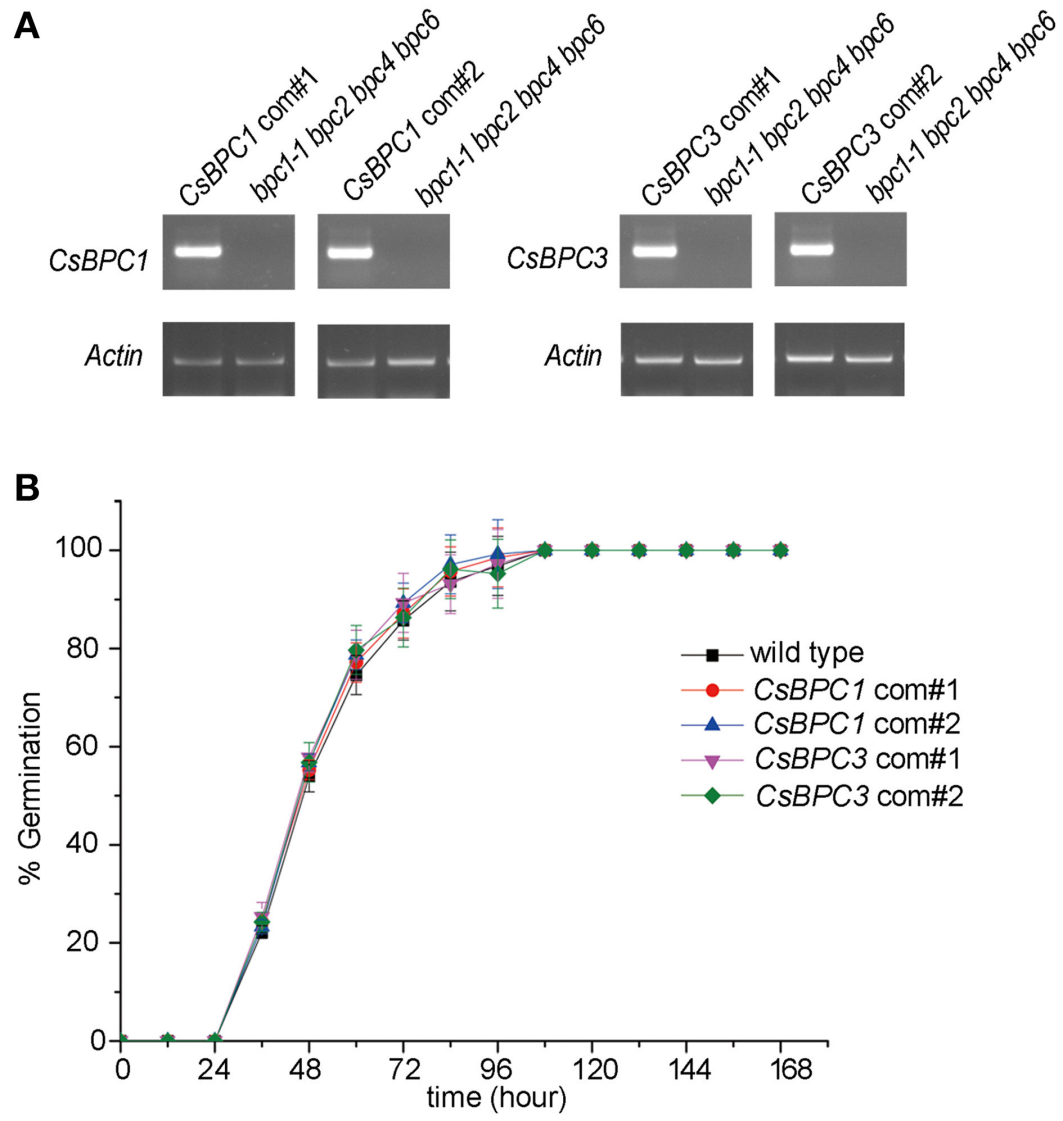
***CsBPC*s could complement the germination phenotype of the *Arabidopsis bpc1-1 bpc2 bpc4 bpc6* mutant. (A)** Expression of the *CsBPC*s were confirmed by RT-PCR. Genomic DNA of the *bpc1-1 bpc2 bpc4 bpc6* mutant was used as negative control. The PCR primers used for detecting *CsBPC1, CsBPC3*, and *Actin* (control) genes are listed in Table S1. **(B)** Seed germination rates of *CsBPC1* com#1, *CsBPC1* com#2, *CsBPC3* com#1, *CsBPC3* com#3, and wild-type *Arabidopsis*. Germination rates (%) of the seeds were analyzed at the indicated time points. The data represent means ± SD of three independent replicates with at least 50 seeds counted per replicate. Values that differed significantly from wild type are indicated. Bonferroni post hoc test detected no significant difference in germination rates between the wild type and complementation of the *bpc1-1 bpc2 bpc4 bpc6* mutant.

To investigate whether the CsBPCs function in germination, *Arabidopsis* lines *CsBPC1*-OX and *CsBPC3*-OX overexpressing *CsBPC1* and *CsBPC3* (Figure S2), respectively, were constructed. The seed germination rate increased in *CsBPC1*-OX and *CsBPC3*-OX lines (*CsBPC1*-OX#1, *CsBPC1*-OX#4, *CsBPC3*-OX#1, and *CsBPC3*-OX#3) compared with wild-type *Arabidopsis* (Figure 1B and Figure S3). We next investigated if cucumber CsBPCs can rescue the late germination phenotype of the *Arabidopsis* quadruple mutant *bpc1-1 bpc2 bpc4 bpc6*. CsBPC1 and CsBPC3, driven by the cauliflower mosaic virus 35S promoter, was transferred into *Arabidopsis bpc1-1 bpc2 bpc4 bpc6*. The resulting transgenic lines *CsBPC1 com*#1, *CsBPC1 com*#2, *CsBPC3 com*#1, and *CsBPC3 com*#2 in which the CsBPCs were detected by RT-PCR (Figure 3A) were selected for the germination experiments. The results showed the germination rate of the complement lines was similar to that of wild type (Figure 3B).

**Figure 4:**
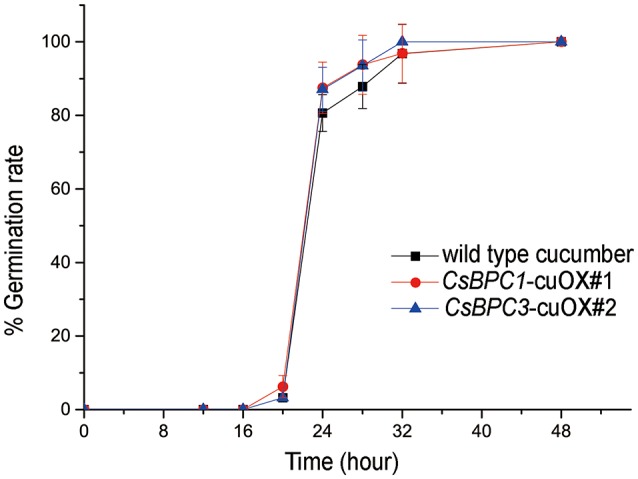
**Germination rate of cucumber**. The seed germination rates of wild-type cucumber and overexpression lines *CsBPC1*-cuOX#1 and *CsBPC3*-cuOX#2. Germination rates (%) were analyzed at the indicated time points. The data represent means ± SD of three independent replicates with at least 50 seeds counted for each replicate. Bonferroni post hoc test detected no difference between the wild-type and the overexpression lines.

To determine whether the CsBPCs function similarly in cucumber, overexpression lines of the CsBPC1 and CsBPC3 in cucumber were constructed. We obtained 9 independent lines for *CsBPC1*-cuOX, and 9 lines for *CsBPC3-*cuOX. Quantitative RT-PCR (qRT-PCR) was used to measure the expression level of CsBPC1 and CsBPC3, respectively, in *CsBPC1*-cuOX and *CsBPC3-*cuOX lines. Two lines for each with the highest expression level were selected (two- to three- fold higher compared with wild-type cucumber; Figure S4). *CsBPC1*-cuOX#1, *CsBPC1*-cuOX#3, *CsBPC3*-cuOX#2, and *CsBPC3*-cuOX#8 were therefore used to assess germination rates. This experiment showed that the increase in germination rates of these overexpression lines was not significant (Figure 4 and Figure S5).

### CsBPCs act upstream of CsABI3

**Figure 5:**
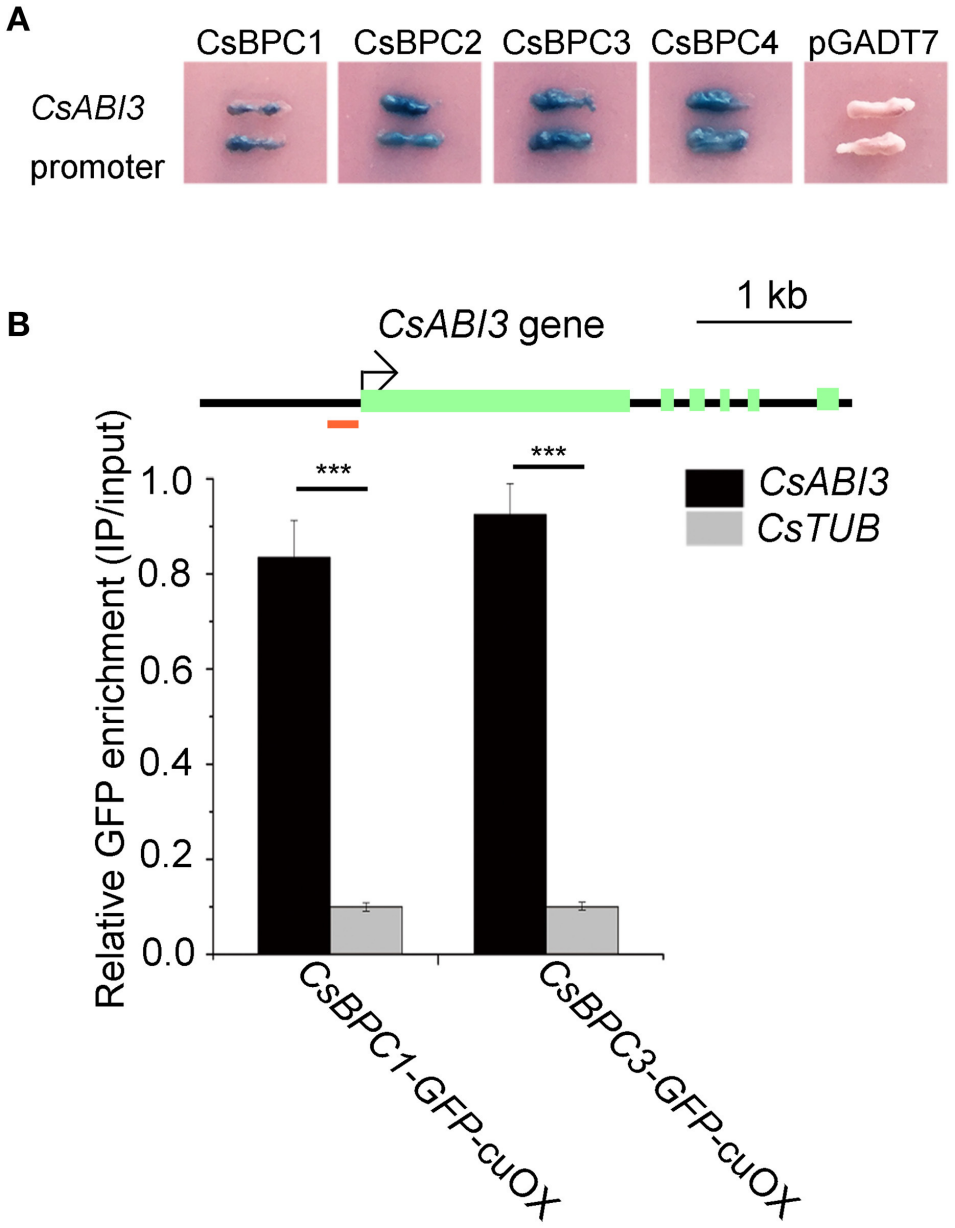
**CsBPC proteins bind to the *CsABI3* promoter. (A)** Yeast one-hybrid assay of interactions between CsBPCs and the *CsABI3* promoter. The *CsABI3* promoter was fused to the pLacZi plasmid that was then linearized and integrated into the genome of the YM4271 yeast strain. Four *CsBPC* genes were cloned into pGADT7 to obtain the CsBPC1/2/3/4-pGAD plasmids. The CsBPCs-pGAD plasmids were transformed into a YM4271 clone harboring the *CsABI3* promoter. The transformants were grown on SD/-Ura-Leu dropout plates containing X-gal (5-bromo-4-chloro-3-indolyl-β-D-galactopyranoside) for white/blue screening. Unmodified PGADT7 was used as a negative control. **(B)** A ChIP assay showed that the CsBPCs bound to the *CsABI3* promoter *in vivo*. GFP enrichment is expressed relative to the chromatin input. One pool comprising three independent transgenic lines was analyzed for each construct. Genomic DNA was obtained via ChIP from cucumber transgenic lines over-expressing CsBPC-GFP fusion proteins (Cs*BPC1*/*3*-*GFP*-cuOX) using an anti-GFP antibody and was then subjected to qPCR analysis. The fragment used for qPCR is indicated by a red bar in the schematic diagram of the *CsABI3* promoter. A control experiment with the *Tubulin* gene was used to establish the ChIP specificity. The mean values ± SD are representative of three independent experiments. ^***^p < 0.001 by Bonferroni *post-hoc* test.

To explore the mechanism by which CsBPCs regulate germination rates, the gene sequence of the *Arabidopsis* germination-related gene *ABI3* was used to search the cucumber genome. We found that the promoter sequence of a cucumber *ABI3* homolog Csa7M336510.1, named *CsABI3*, showed high similarity (54.6%) to that of *Arabidopsis ABI3*. The promoter of *CsABI3* contains three typical 9 bp DNA consensus sequences (RGARAGRRA) that comprise the BPC protein binding motif (Kooiker et al., 2005; Figure S6). The first *cis*-regulatory element is composed of 9 nucleotides (5′-AAAGAGAGA-3′) and is located 303 bp upstream of the translational start codon; the second *cis*-regulatory element, which consists of 16 nucleotides (5′-CTTCTTTCTCTTTCCT-3′), is located 279 bp upstream of the translational start point (inverted 5′-AGGAAAGAGAAAGAAG-3′); the third element, consisting of 9 nucleotides (5′-AAAGAAAGA-3′), is found 13 bp upstream of the translational start point. To determine whether *CsABI3* is targeted by the CsBPCs, a yeast one-hybrid assay was performed which showed that all four CsBPCs interacted with the *CsABI3* promoter (Figure 5A). Furthermore, a ChIP assay was performed to confirm the binding ability of the CsBPC1 and CsBPC3 to the *CsABI3* promoter. Results showed that *CsABI3* promoter fragments were enriched in anti-GFP samples compared with the ChIP samples prepared using *Tubulin* controls (Figure 5B).

### CsBPCs act as negative regulators of CsABI3

**Figure 6:**
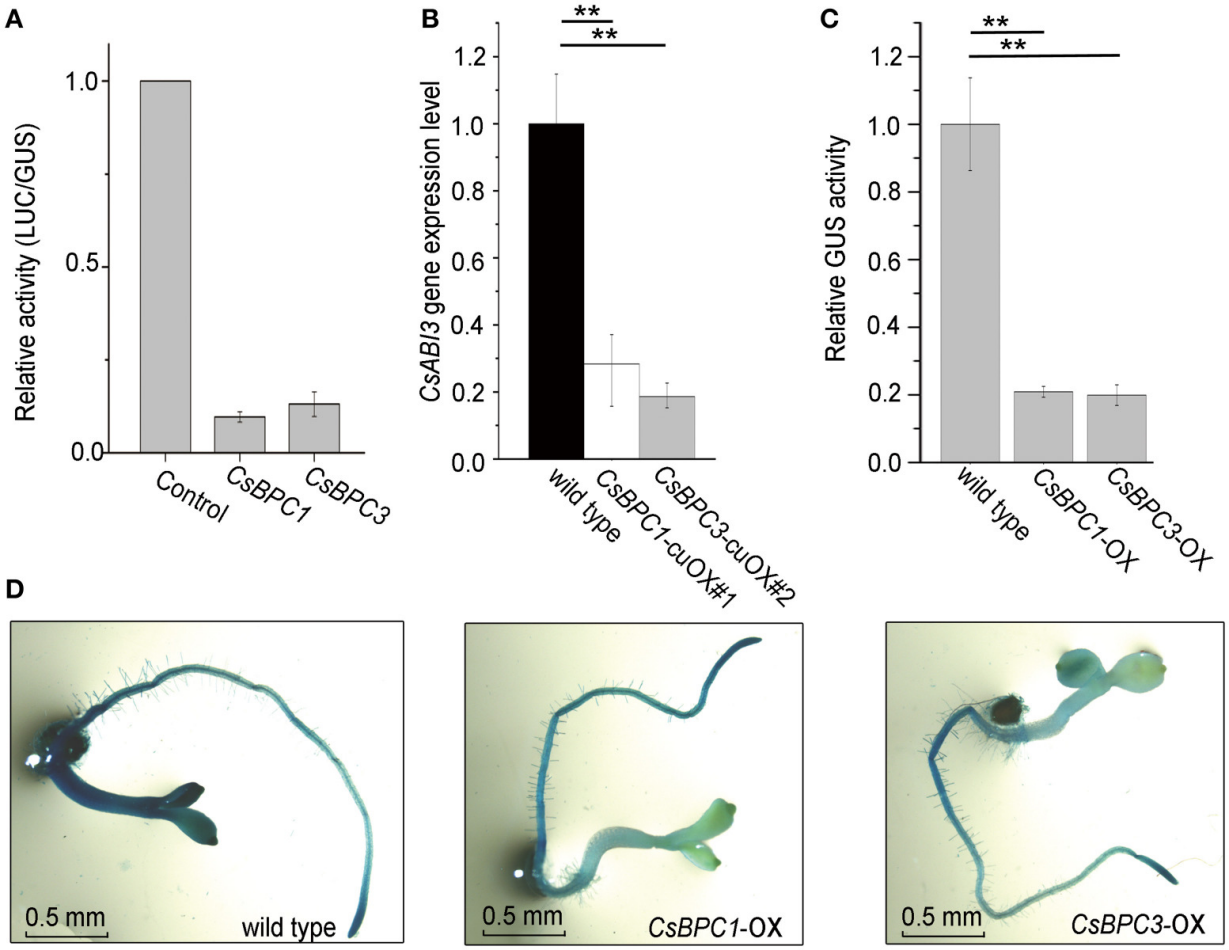
**CsBPCs act as negative regulators of *CsABI3*. (A)** LUC activity in a tobacco leaves transiently expressing the *CsABI3* promoter co-infiltrated with empty control plasmid (CK) or plasmids containing the *CsBPC*s genes fused to the *35S* promoter. Data shown are averages ± *SD* (*n* = 3). **(B)** *CsABI3* mRNA levels in *CsBPC1-*cuOX#1, *CsBPC3-*cuOX#2, and wild-type cucumber seedlings grown for 4 d. Data shown are averages ± *SD* (*n* = 3). Three biological replicates were included for each experiment and 10 seedlings were included for each line. ^**^p < 0.01 by Bonferroni post hoc test. **(C)** Quantitation of GUS activity of *ProABI3*:*GUS* in wild-type, *CsBPC1-*OX, and *CsBPC3-*OX *Arabidopsis* seedlings grown for 4 d. The mean values ± *SD* are representative of three independent experiments. ^**^p < 0.01 by Bonferroni post hoc test. **(D)** Histochemical GUS staining for GUS activity shown in **(C)**. A similar trend was observed in each of the three biological repeats, with representative images shown here.

The interactions between the CsBPCs and the *CsABI3* promoter were further investigated by evaluating the capacity of the CsBPCs to regulate the *CsABI3* promoter. We developed a transient transcription assay system in which tobacco leaves were cotransfected with a *35S:CsBPC1/3* effector plasmid and a reporter construct carrying a fusion between the *CsABI3* promoter and *firefly luciferase* (*fLUC*) that acted as a reporter for transcriptional activation (Figure 6A). The results showed that CsBPC1 and CsBPC3 resulted in a reduction in fLUC levels to about 10% of the controls, suggesting a negative relationship between the CsBPCs and *CsABI3*. Consistent with this negative relationship, *CsABI3* mRNA levels were significantly lower in the cucumber CsBPCs overexpression lines (*CsBPC1*-cuOX#1 and *CsBPC3-*cuOX#2) compared with wild-type cucumber (Figure 6B). A similar result was found in the *CsBPC1-*cuOX#3 and *CsBPC3-*cuOX#8 lines (data not shown). Furthermore, overexpression of both *CsBPC1* and *CsBPC3* decreased the level of *GUS*expression driven by the 1.0-kb fragment of the *CsABI3* promoter (*ProCsABI3*:*GUS*) (Figures 6C,D).

### The interaction between CsBPCs and CsABI3 during germination

**Figure 7:**
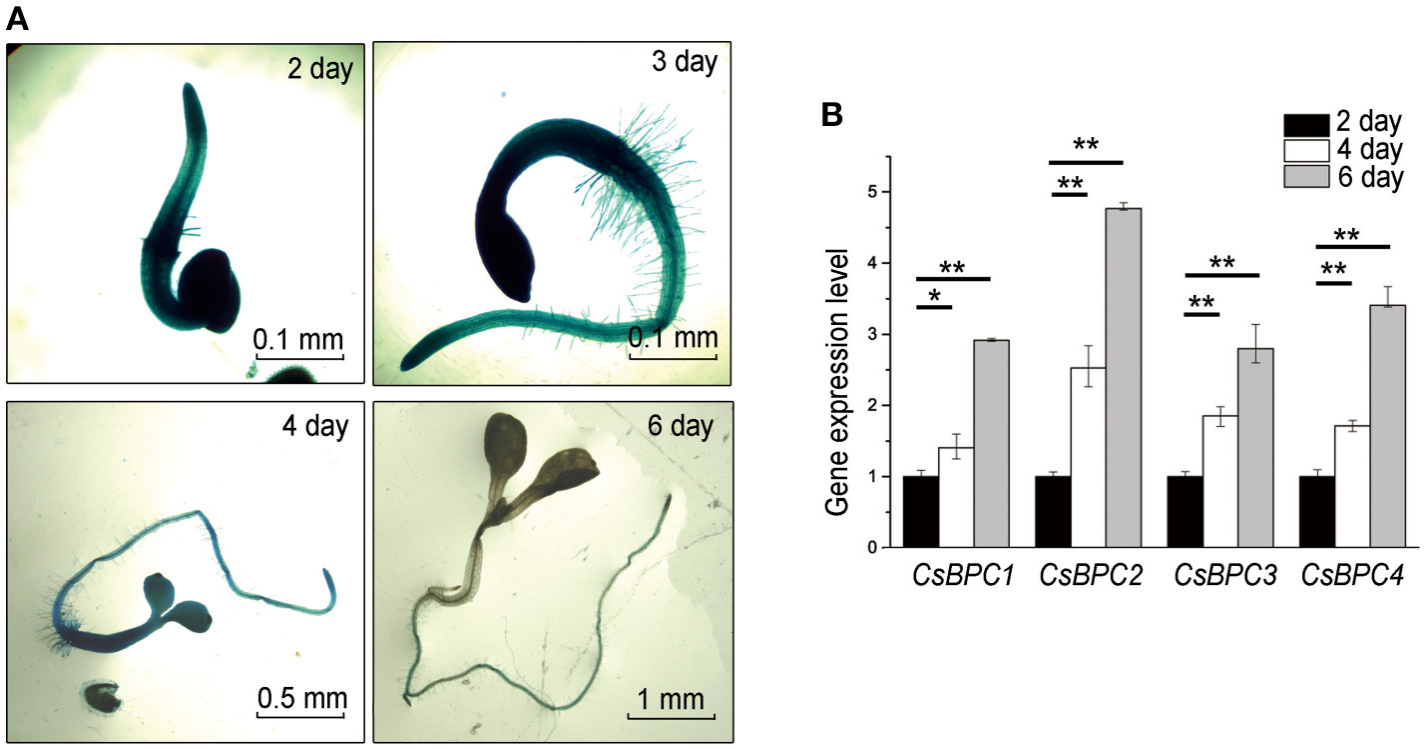
**Expression of *CsABI3* and the *CsBPCs*. (A)** Expression of *ProABI3*:*GUS* in wild-type *Arabidopsis* seedlings grown for different periods of time. A similar trend was observed in each of three biological repeats, with representative images shown here. **(B)** Expression of the *CsBPCs* in cucumber seedlings grown for different periods of time. RNA was extracted and the expression levels of the *CsBPCs* were analyzed by qRT-PCR. Data shown are averages ± SD (*n* = 3). Three biological replicates were included for each experiment and 10 seedlings were included for each line. ^*^p < 0.05 and ^**^p < 0.01 by Bonferroni post hoc test.

To validate the regulatory role of CsBPCs on *CsABI3* in seed germination, the level of *GUS* expression driven by the *CsABI3* promoter was observed. *CsABI3* promoter activity was high for 2 d after germination before dropping quickly over the following days to a barely detectable level 6 d after germination (Figure 7A). Conversely, the expression levels of the *CsBPC*s increased gradually in cucumber from 2–6 d after germination (Figure 7B). These results are consistent with *CsABI3* expression being negatively regulated by the four CsBPC transcription factors in the germination and early development period.

**Figure 8:**
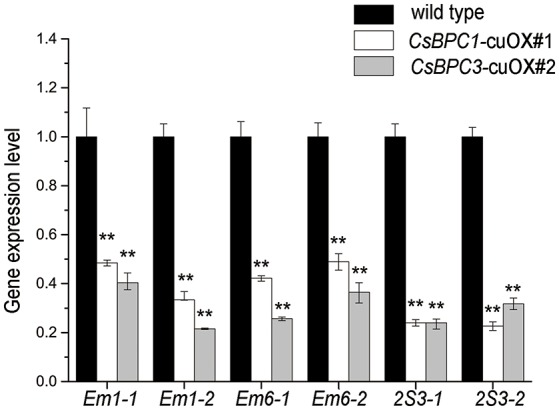
**Overexpression of the *CsBPCs* reduces the expression of cucumber homologs of seed maturation genes**. The expression levels of cucumber homologs of *Em1, Em6*, and *2S3* were examined in *CsBPC1*-cuOX#1, *CsBPC3*-cuOX#2, and wild-type cucumber plants. RNA was extracted from seedlings grown for 3 d and the expression levels were analyzed by qRT–PCR. Data shown are averages ± *SD* (*n* = 3). Three biological replicates were included for each experiment and 10 seedlings were included for each line. Values that differed significantly from wild type are indicated. ^**^ *p* < 0.01 by Bonferroni *post-hoc* test.

ABI3 is an essential embryogenesis factor (Giraudat et al., 1992; Finkelstein et al., 1998) and is particularly important for late embryo development that takes place after embryonic cell division and morphogenesis are complete (Mccourt, 2003). The *Arabidopsis* seed maturation genes *Em1, Em6*, and the 2S *ALBUMIN* 3 (*2S3*) are all regulated by ABI3 (Parcy et al., 1994; Carles et al., 2002). Considering the interactions between the CsBPCs and *CsABI3*, it was reasonable for us to speculate that the CsBPCs might be involved in ABI3-mediated regulation during seed germination. To validate this possibility, we conducted a database search and identified a number of cucumber homologs of these *Arabidopsis* seed maturation genes including Csa3M078820.1, named *cu2S3-1*; Csa3M080340.1, named *cu2S3-2*; Csa4M311730.1, named *CuEm1-1*; Csa4M311230.1, named *CuEm1-2*; Csa4M311230.1, named *cuEm6*-1; and Csa4M311730.1, named *cuEm6-2*. qRT-PCR results showed that the expression of each of these genes was reduced in the cucumber *CuBPC1/3* overexpression lines, strongly suggesting a role for the CsBPCs in the ABI3-mediated seed germination (Figure 8). Similar expression patterns were observed in the *CsBPC1*-cuOX#3 and *CsBPC3*-cuOX#8 lines (data not shown).

## Discussion

The breaking of seed dormancy and the beginning of autotrophic growth is a critical transition for seed plants. During this process, plants must monitor the environment and respond properly. The mechanism underlying seed dormancy and the transition to autotrophic growth requires further characterization. In this study, the role of cucumber CsBPCs during germination was investigated.

Cucumber, a well-known vegetable that is grown worldwide, displays shallow seed dormancy that often results in vivipary and causes serious damage to cucumber seed production and quality (Cao et al., 2016). Unfortunately, few published studies have focused on vivipary in cucumber. Previously, we found that the seeds of an *Arabidopsis* quadruple mutant with mutations in four *BPC* genes had significantly reduced germination rates (Figure 1A). To investigate whether the homologous cucumber *CsBPCs* have a similar function, *CsBPC-*overexpressing lines were generated showing that increased *CsBPC* expression resulted in greater seed germination rates in *Arabidopsis* (Figure 1B and Figure S3). Furthermore, cucumber CsBPCs functionally complement the germination phenotype of *Arabidopsis bpc1-1 bpc2 bpc4 bpc6* mutants (Figure 3).

However, the effect of *CsBPC* overexpression on seed germination was not significant in cucumber (Figure 4 and Figure S5). While the enhancement of gene expression driven by the CaMV 35S promoter is efficient in *Arabidopsis* (Shin et al., 2007), the expression levels of the *CsBPCs* in transgenic cucumber lines increased by only two- to three- fold compared with those of wild-type cucumber (Figure S1) which is likely to result in the unaffected germination rate of these cucumber seeds. Thus, a more effective mrthod such as the CRISPR/Cas9 system must be established in the future to enhance gene expression levels in cucumber. These results show that cucumber CsBPCs are vital for the regulation of seed germination. Additionally, previous studies have shown that the ovules of the *Arabidopsis BPC* quadruple mutant *bpc1-1 bpc2 bpc4 bpc6* had excess cell layers in the inner integument, suggesting that *BPCs* were also involved in seed development (Monfared et al., 2011).

Given that CsBPCs affect seed germination, we aimed to examine the molecular mechanism underlying their regulation of this process. Consistent with previous reports that *Arabidopsis* BPCs bind specifically to GA-rich domains (RGARAGRRA; Kooiker et al., 2005), three GA-rich domains were found in the *CsABI3* promoter that may represent the BPC binding motif (Figure S6). In animals, such GAGA-motif containing DNA is bound by GAGA factors (GAFs) that play a role in both the repression and activation of homeobox genes through their interactions with Polycomb (PcG) and trithorax group (trxG) proteins, and thus contributes to a wide range of development events (Strutt et al., 1997; Francis and Kingston, 2001; Lehmann, 2004; Muller and Kassis, 2006; Schuettengruber et al., 2009; Berger and Dubreucq, 2012). In contrast to chromatin factors PcG and TrxG that are well-conserved between animals and plants, there are no homologs of the animal GAFs in plants (Strutt et al., 1997; Francis and Kingston, 2001; Lehmann, 2004; Muller and Kassis, 2006; Schuettengruber et al., 2009; Berger and Dubreucq, 2012). Given the functional similarities between BPCs and GAFs, BPCs were suggested to be GAF substitutes in plants (Berger et al., 2011; Berger and Dubreucq, 2012; Simonini et al., 2012). Nevertheless, a GAGA element in the *LEC2* gene promoter bound by BPC1 was not required for the activity of PcG or TrxG, suggesting that BPCs might have other unknown functions (Berger et al., 2011).

*ABI3*, the candidate target gene of the CsBPC proteins, has been known as an essential regulator of seed dormancy and germination (Parcy et al., 1994; Nambara et al., 2000; Lopezmolina et al., 2002; Bassel et al., 2006; Tamura et al., 2006; Park et al., 2011; Feng et al., 2014). ABI3 regulates a substantial number of seed development-related genes and, therefore, functions primarily in the establishment of seed dormancy (Parcy et al., 1994; Nambara et al., 2000; Lopezmolina et al., 2002; Nakashima et al., 2006). During seed germination, *ABI3* expression levels remain relatively high (Figure 7A; Park et al., 2011). Despite the fact that *ABI3* gene expression is regulated by transcription factors like FUS2, LEC2, LEC3 (To et al., 2006), and RAV1 (Feng et al., 2014), WRKY41(Ding et al., 2014) is the only known upstream regulator that continues to regulate *ABI3* expression at seed germination and over the following days (Ding et al., 2014).

The identification of unknown upstream regulators of *ABI3* in the seed germination pathway is vital for us to understand the molecular mechanisms underlying this process. Here, we used yeast one-hybrid assays to demonstrate that the *CsABI3* promoter was bound by the four cucumber CsBPCs, CsBPC1–4 (Figure 5A). Additionally, ChIP assays confirmed that CsBPC1 and CsBPC3 (representing Class I and Class II BPCs, respectively) could bind directly to the *CsABI3* promoter (Figure 5B). Having shown that the cucumber CsBPCs act upstream of *CsABI3*, we explored the relationship between the CsABI3 and CsBPCs further. Analyses of the *CsABI3* promoter and CsBPC proteins *in vivo* showed that the overexpression of either *CsBPC1* or *CsBPC3* resulted in decrease of ~90%in the activity of the *CsABI3* promoter (Figure 6A). The regulatory functions of the CsBPCs were further confirmed in transgenic cucumber plants overexpressing *CsBPC1* or *CsBPC3* fused to *GFP*. The qRT–PCR analysis showed that expression of *CsABI3* was reduced in these lines compared with wild-type cucumber (Figure 6B). Consistent with these results, GUS activity driven by the *CsABI3* promoter was significantly reduced in *Arabidopsis* lines overexpressing *CsBPC1* or *CsBPC3* (Figures 6C,D). The negative relationship between the CsBPCs and the *CsABI3* promoter, together with the ability of the BPCs to bind to the *CsABI3* promoter, suggests that CsBPCs might be involved in the negative regulation of *CsABI3*.

Having shown that cucumber CsBPCs are involved in seed germination and that CsBPCs act upstream of CsABI3, it is reasonable for us to speculate that the CsBPCs affect seed germination via the CsABI3 pathway.

Previous studies have shown that *Em1, Em6*, and *2S3* are representative seed maturation genes that are controlled by the ABA pathway through ABI3 (Parcy et al., 1994). The qRT-PCR assays showed that, in 3-day-old transgenic cucumber lines, the overexpression of either *CsBPC1* or *CsBPC3* significantly reduced the expression levels of *Em1, Em6*, and *2S3* homologs in cucumber (Figure 8), supporting a role for CsBPCs upstream of *CsABI3* in seed germination. It has also been suggested that there are more putative BPC target genes in the plant kingdom. Sequence analysis identified more than 12,000 *Arabidopsis* genes containing more than one GA-rich stretch within their regulatory regions (Santi et al., 2003; Berendzen et al., 2006; Deng et al., 2013; Hecker et al., 2015), suggesting that BPCs might act on a variety of target genes in diverse developmental processes. The significance of the relatively high number of GAGA-binding factors in the plant kingdom indicates the importance of the BPC family in growth and development processes.

Moreover, a previous study showed that LEC2, a positive regulator of ABI3, controlled most aspects of seed maturation (To et al., 2006). *Arabidopsis* BPCs were reported to act redundantly to regulate *LEC2* expression through its specific GAGA cis-regulatory motif (Berger et al., 2011). This implies that the BPCs might also be involved in the ABI3 pathway indirectly via *LEC2* regulation. In the present study, we describe four cucumber CsBPC transcription factors, CsBPC1, CsBPC2, CsBPC3, and CsBPC4, that are direct upstream regulators of *CsABI3*. Furthermore, we suggest that by regulating *CsABI3*, CsBPCs control seed germination. Further, investigation is needed to characterize the precise mechanism regulating the CsBPC-CsABI3 pathway controlling seed germination.

## Author contributions

Substantial contributions to the conception or design of the work; or the acquisition, analysis, or interpretation of data for the work (YM, YML, LB, SL, CH, YY, XY, and YSL); Drafting the work or revising it critically for important intellectual content (YM, YML, LB, SL, CH, YY, XY, and YSL); Final approval of the version to be published (YM, YML, LB, SL, CH, YY, XY, and YSL); Agreement to be accountable for all aspects of the work in ensuring that questions related to the accuracy or integrity of any part of the work are appropriately investigated and resolved (YM, YML, LB, SL, CH, YY, XY, and YSL).

## Funding

Sincere thanks for the funding support provided by the Earmarked fund for Modern Agro-industry Technology Research System (CARS-25-C-01), Science and Technology Innovation Program of the Chinese Academy of Agricultural Sciences (CAAS-ASTIP-IVFCAAS) and the support by the Key Laboratory of Horticultural Crop Biology and Germplasm Innovation, Ministry of Agriculture, China. The funders had no role in study design, data collection and analysis, decision to publish, or preparation of the manuscript.

### Conflict of interest statement

The authors declare that the research was conducted in the absence of any commercial or financial relationships that could be construed as a potential conflict of interest.
