# Supplementary material for: Cucumber CsBPCs Regulate the Expression of CsABI3 during Seed Germination
Source: Front Plant Sci. 2017 Apr 3;8:459. doi: 10.3389/fpls.2017.00459 (PMC5376566; doi:10.3389/fpls.2017.00459)
Supplement: Supplementary file 1 [file Table1.DOCX]

**Supplementary Table 1.** Primers used in this study

| Primer Name | Primer Sequence (5’ to 3’) | Experiment |
| --- | --- | --- |
| CsBPC1-BamHI-2300-S | CGCGGATCCATGGATGACGATGCGTTAAA | Plant Transformation |
| CsBPC1-SpeI-2300-A | CTAGACTAGTCCTGATTGTGACAAACT |  |
| CsBPC3-BamHI-2300-S | CGCGGATCCATGGATGATGGCCGTCAACA |  |
| CSBPC3-SpeI-2300-A | CTAGACTAGTCCTGATTGTTATGTAGC |  |
| CsBPC1-XhoI-PBSK-S | CCGCTCGAGATGGATGACGATGCGTTAAA | GFP Assays |
| CsBPC1-EcoRI-PBSK-A | CCGGAATTCACCTGATTGTGACAAACT |  |
| CsBPC2-XhoI-PBSK-S | CCGCTCGAGATGGATGGTGATGCTTTGAA |  |
| CsBPC2-EcoRI-PBSK-A | CCGGAATTCACCTGATTGTGACAAACT |  |
| CsBPC3-XhoI-PBSK-S | CCGCTCGAGATGGATGATGGCCGTCAACA |  |
| CsBPC3-EcoRI-PBSK-A | CCGGAATTCACCTGATTGTTATGTAGC |  |
| CsBPC4-XhoI-PBSK-S | CCGCTCGAGATGGATGACAGTGGACACCG |  |
| CsBPC4-EcoRI-PBSK-A | CCGGAATTCACTTGATGGTGATGTAAC |  |
| ProCsABI3-HindIII-LUC-S | CCCAAGCTTGGGCACCGTTGTTAGCCACC | GUS Staining |
| ProCsABI3-NcoI-LUC-A | CATGCCATGGGTTCCTTTACAAACCCTGCAT |  |
| CsABI3P-KpnI-lac-S | CGGGGTACCGGGCACCGTTGTTAGCCACCA | Yeast One-Hybrid |
| CsABI3P-SalI-lac-A | ACGCGTCGACTTCCTTTACAAACCCTGC |  |
| CsBPC1-EcoRI-AD-S | CCGGAATTCATGGATGACGATGCGTTAAA |  |
| CsBPC1-XhoI-AD-A | CCGCTCGAGCTACCTGATTGTGACAAACT |  |
| CsBPC2-EcoRI-AD-S | CCGGAATTCATGGATGGTGATGCTTTGAA |  |
| CsBPC2-XhoI-AD-A | CCGCTCGAGCTACCTGATTGTGACAAACT |  |
| CsBPC3-EcoRI-AD-S | CCGGAATTCATGGATGATGGCCGTCAACA |  |
| CsBPC3-XhoI-AD-A | CCGCTCGAGCTACCTGATTGTTATGTAGC |  |
| CsBPC4-EcoRI-AD-S | CCGGAATTCATGGATGACAGTGGACACCG |  |
| CsBPC4-XhoI-AD-A | CCGCTCGAGCTACTTGATGGTGATGTAAC |  |
| ProCsABI3-HinIII-LUC-S | CCCAAGCTTGGGCACCGTTGTTAGCCACC | LUC Activity Assay |
| ProCsABI3-HinIII-LUC-A | CGCGGATCCTTCCTTTACAAACCCTGCAT |  |
| CsABI3-qPCR-S | TGCTGCTGATCGCTCTAATTCCTC | qRT-PCR |
| CsABI3-qPCR-A | TCTTCTCTGGTTTCCATCCCTGTC |  |
| CsBPC1-qPCR-S | TGAAGAAGGAAGGTGGGAAAG |  |
| CsBPC1-qPCR-A | GAACAGCCGTGCTAGTATCTT |  |
| CsBPC2-qPCR-S | ACCTGTGAAGAAGGGAACTAAAC |  |
| CsBPC2-qPCR-A | TAGGCTTTCGAGGCTTCTTTG |  |
| CsBPC3-qPCR-S | GGAAAGCGAGCGAAGGATAA |  |
| CsBPC3-qPCR-A | GTGGCAGCATGTCTATTCAAATC |  |
| CsBPC4-qPCR-S | GTCCCAACACCAAACAAGAAAG |  |
| CsBPC4-qPCR-A | TCGTCACCTGCACTCATTATAC |  |
| CsTUB-qPCR-S | ACGCTGTTGGTGGTGGTAC |  |
| CsTUB-qPCR-A | GAGAGGGGTAAACAGTGAATC |  |
| Cs2S3-1S | CTAGTTTCCATCCTCCTCCTTTC |  |
| Cs2S3-1A | CGTTGGCTTTGGTTGTCATC |  |
| Cs2S3-2S | TGCCATGGAAGAACAGAGAAG |  |
| Cs2S3-2A | ATACCGCACATGGATGGTAAG |  |
| CsEm1-1S | GCCAAGAAGGCTACAAAGAGA |  |
| CsEm1-1A | CTCATCGATCTCGATTCCTTCC |  |
| CsEm1-2S | GACACGAAGGATACCAAGAGATG |  |
| CsEm1-2A | TCGTCAATCTCAACACCTTCC |  |
| CsEm6-1S | GACACGAAGGATACCAAGAGATG |  |
| CsEm6-1A | TCGTCAATCTCAACACCTTCC |  |
| CsEm6-2S | GCCAAGAAGGCTACAAAGAGA |  |
| CsEm6-2A | CTCATCGATCTCGATTCCTTCC |  |
| CsBPC1-PCR-S | CTCTTCGTCGAGGGATGAAATAG | RT-PCR |
| CsBPC1-PCR-A | TCACTCATGGGCAAAGGATAAG |  |
| CsBPC3-PCR-S | GAAGAAGGTTGGCGAAGATTTG |  |
| CsBPC3-PCR-A | TACGAGCATGGCGTTTGT |  |
| Actin-PCR-S | CAGGCCGTTCTTTCTCTCTATG |  |
| Actin-PCR-A | CTCTCAGCTCCGATGGTTATG |  |
| CHIP-CsABI3-F | GATTTCCCTCCACTTCCTGATT | CHIP |
| CHIP-CsABI3-R | GAAGACGAGGATGACGATGAC |  |
| CsTUB-F | ACGCTGTTGGTGGTGGTAC |  |
| CsTUB-R | GAGAGGGGTAAACAGTGAATC |  |
