# Supplementary material for: Cucumber CsBPCs Regulate the Expression of CsABI3 during Seed Germination
Source: Front Plant Sci. 2017 Apr 3;8:459. doi: 10.3389/fpls.2017.00459 (PMC5376566; doi:10.3389/fpls.2017.00459)
Supplement: Supplementary file 2 [file Image1.PDF]

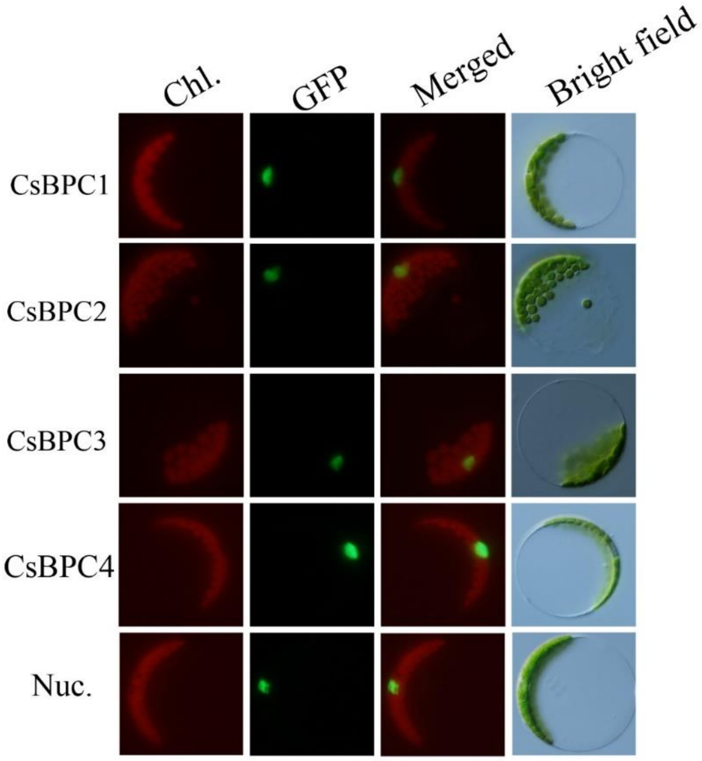

Figure S1. Localization of a nucleus-targeted CsBPCs-GFP in *Arabidopsis*.

35S-GFP transiently expressed in an *Arabidopsis* protoplast. The 35S:CsBPC1-GFP, 35S:CsBPC2-GFP, 35S:CsBPC4-GFP and 35S:CsBPC4-GFP constructs were transformed into *Arabidopsis* protoplasts, and localization of fusion proteins was visualized by confocal microscopy. Nuc., signal of nuclear localization protein, fibrillarin; A representative image from a single protoplast is shown.
