# Supplementary material for: Cucumber CsBPCs Regulate the Expression of CsABI3 during Seed Germination
Source: Front Plant Sci. 2017 Apr 3;8:459. doi: 10.3389/fpls.2017.00459 (PMC5376566; doi:10.3389/fpls.2017.00459)
Supplement: Supplementary file 3 [file Image2.PDF]

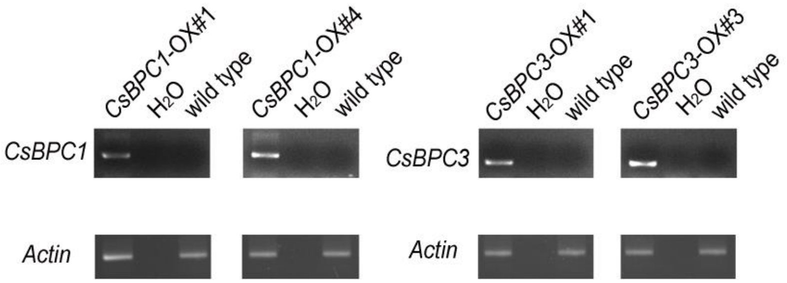

Figure S2. PCR analysis of Arabidopsis lines overexpressing *CsBPC1* and *CsBPC3*.

Expression of the transgenes were confirmed by RT-PCR. H_2O and wild-type DNA were used as negative control. The PCR primers used for detecting *CsBPC1*, *CsBPC3* and *Actin* (control) genes are listed in Supplementary Table 1.
