# Supplementary material for: Cucumber CsBPCs Regulate the Expression of CsABI3 during Seed Germination
Source: Front Plant Sci. 2017 Apr 3;8:459. doi: 10.3389/fpls.2017.00459 (PMC5376566; doi:10.3389/fpls.2017.00459)
Supplement: Supplementary file 4 [file Image3.pdf]

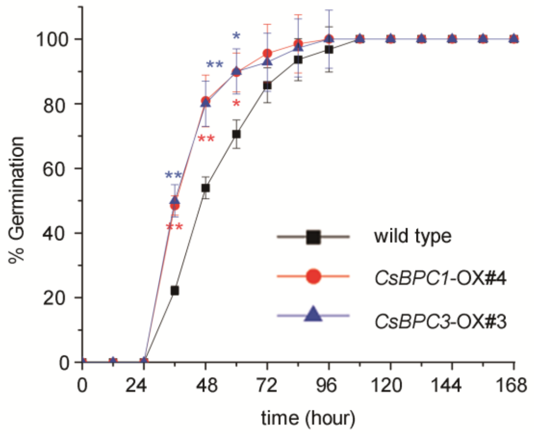

Figure S3. Seed germination rate of *CsBPC1*-OX#4, *CsBPC3*-OX#3 and wild-type *Arabidopsis*.

Germination rates (%) of the seeds were analyzed at the indicated time points. The data represent means ±SD s of three independent replicates (at least 50 seeds were counted for each repeat). Values significantly different from wild type are indicated. *$p < 0.05$ and **$p < 0.01$ by Bonferroni post hoc test.
