# Supplementary material for: Cucumber CsBPCs Regulate the Expression of CsABI3 during Seed Germination
Source: Front Plant Sci. 2017 Apr 3;8:459. doi: 10.3389/fpls.2017.00459 (PMC5376566; doi:10.3389/fpls.2017.00459)
Supplement: Supplementary file 5 [file Image4.PDF]

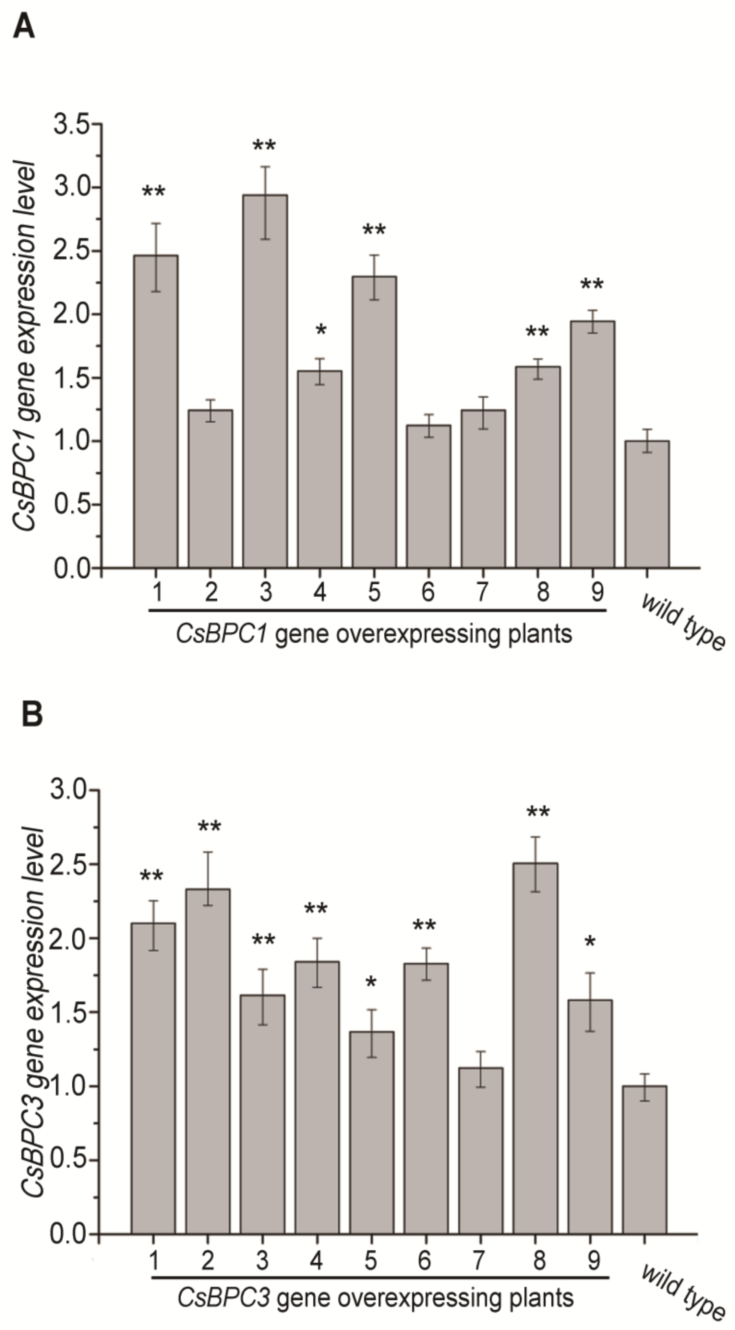

Figure S4. Expression level of the *CsBPC*s mRNA in transgenic cucumber lines grown for 3 d.

The total RNA was extracted from germinating cucumber seeds grown for 3 days and the levels of target transcript were analyzed using RT-qPCR. Experiments were performed in biological triplicate (10 seedlings per line per experiment). Values significantly different from wild type are indicated. *$p < 0.05$ and ** $p < 0.01$ by Bonferroni post hoc test.
