# Supplementary material for: Cucumber CsBPCs Regulate the Expression of CsABI3 during Seed Germination
Source: Front Plant Sci. 2017 Apr 3;8:459. doi: 10.3389/fpls.2017.00459 (PMC5376566; doi:10.3389/fpls.2017.00459)
Supplement: Supplementary file 6 [file Image5.PDF]

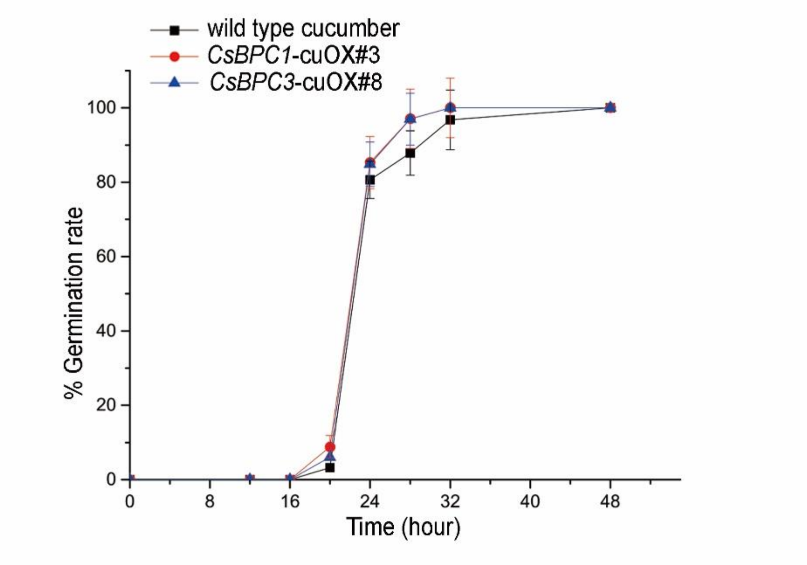

Figure S5. Germination rate of cucumber.

Seed germination rate of wild type cucumber and the overexpression lines *CsBPC*1-cuOX#3 and *CsBPC3*-cuOX#8. Germination rates (%) of the seeds were analyzed at the indicated time points. The data represent means ±SD s of three independent replicates (at least 50 seeds were counted for each repeat). Bonferroni post hoc test detected no difference between the wild-type and the overexpression lines.
