# Supplementary material for: Cucumber CsBPCs Regulate the Expression of CsABI3 during Seed Germination
Source: Front Plant Sci. 2017 Apr 3;8:459. doi: 10.3389/fpls.2017.00459 (PMC5376566; doi:10.3389/fpls.2017.00459)
Supplement: Supplementary file 7 [file Image6.PDF]

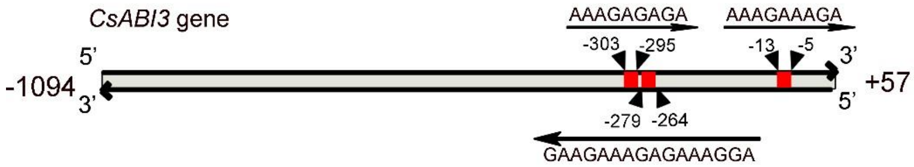


**Figure S6.** Schematic diagram of the Cs*ABI3* promoter.

The GA-rich element is indicated by the red box. Numbers indicate the positions of nucleotides relative to the ATG start codon.
